# Supplementary material for: Endometrial Stromal Senescence Mediates the Progression of Intrauterine Adhesions
Source: Int J Mol Sci. 2025 Apr 28;26(9):4183. doi: 10.3390/ijms26094183 (PMC12071859; doi:10.3390/ijms26094183)
Supplement: Supplementary file 1 [file ijms-26-04183-s001.zip › ijms-3579774-supplementary.pdf]

Supplementary Materials

Figure S1: Patients with IUA and TE are characterized by an increased level of stromal senescence in endometrium during the proliferative phase compared to healthy donors. Normal – healthy donors, TE – patients with thin endometrium, IUA – patients with intrauterine adhesions. Analysis was performed on scRNA-seq datasets PRJNA730360 (Normal n = 3, TE n = 3) and PRJNA784021 (IUA n = 3), integrated and annotated as demonstrated at Figure 2. Evaluation was performed in stromal cells using EnSC-specific senescence gene signatures; Figure S2: Among mesenchymal lineage cell subtypes only stromal cells of patients with IUA exhibit an increased level of senescence during the proliferative phase compared to healthy donors. Normal – healthy donors, IUA – patients with intrauterine adhesions. Analysis was performed on scRNA-seq datasets PRJNA730360 (Normal n = 3) and PRJNA784021 (IUA n = 3), integrated and annotated as demonstrated at Figure 2. (A – D) UMAP representation of mesenchymal lineage cells colored by samples origin, phenotype, cell subtypes, and UCell enrichment scores for the Saul SenMayo geneset, respectively. (E) Violin plots of UCell enrichment scores for the Saul SenMayo geneset for the cell subtypes between Normal donors and patients with IUA; Table S1: Genesets used in the study; Table S2: Functional annotation of genesets used in the study; Table S3: Differential secreted signaling in proliferative endometrium of patients with IUA compared to Normal donors.
