# Supplementary figures and images for: Endometrial Stromal Senescence Mediates the Progression of Intrauterine Adhesions
Source: Int J Mol Sci. 2025 Apr 28;26(9):4183. doi: 10.3390/ijms26094183 (PMC12071859; doi:10.3390/ijms26094183)

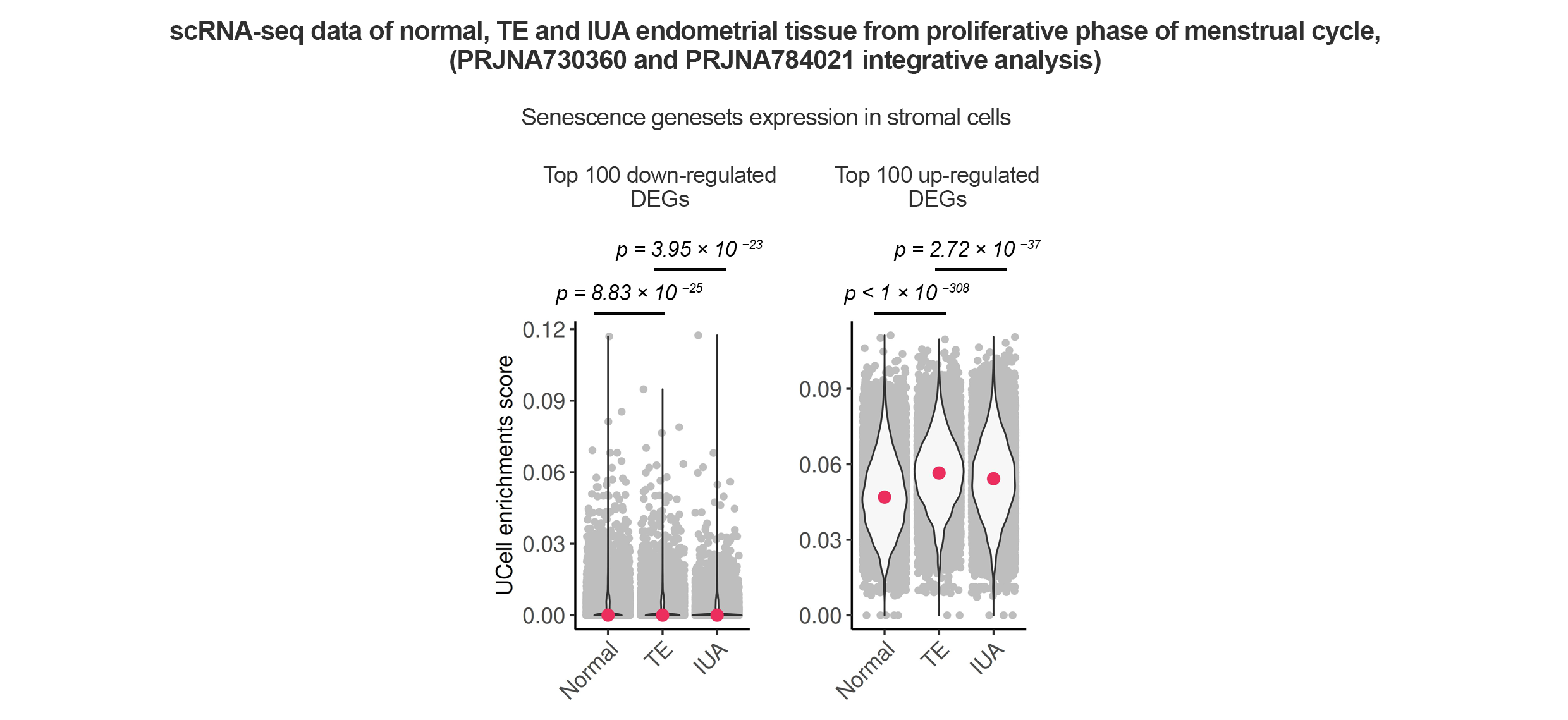

Supplement: Supplementary file 1 [file ijms-26-04183-s001.zip › Supplemental Figure S1.tif]

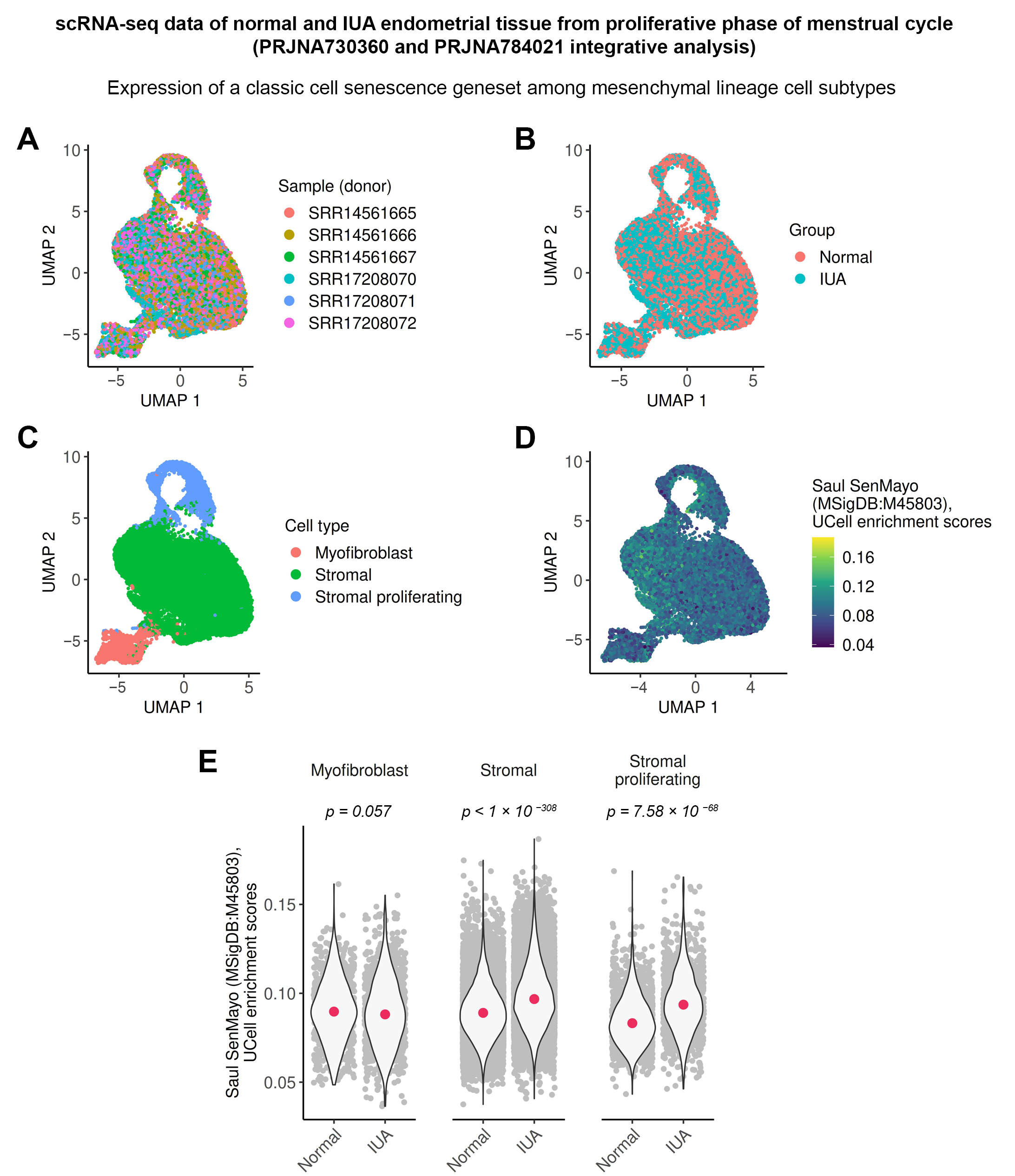

Supplement: Supplementary file 1 [file ijms-26-04183-s001.zip › Supplemental Figure S2.tif]
